# Comparative Study of Chemical Composition and Biological Activity of Yellow, Green, Brown, and Red Brazilian Propolis

**DOI:** 10.1155/2016/6057650

**Published:** 2016-07-21

**Authors:** Christiane Schineider Machado, João Benhur Mokochinski, Tatiana Onofre de Lira, Fátima de Cassia Evangelista de Oliveira, Magda Vieira Cardoso, Roseane Guimarães Ferreira, Alexandra Christine Helena Frankland Sawaya, Antonio Gilberto Ferreira, Cláudia Pessoa, Osmany Cuesta-Rubio, Marta Chagas Monteiro, Mônica Soares de Campos, Yohandra Reyes Torres

**Affiliations:** ^1^Departamento de Química, Universidade Estadual do Centro-Oeste (UNICENTRO), 85040-080 Guarapuava, PR, Brazil; ^2^Departamento de Biologia Vegetal, Instituto de Biologia, Universidade Estadual de Campinas (UNICAMP), 13083-862 Campinas, SP, Brazil; ^3^Departamento de Química, Universidade Federal de São Carlos (UFSCar), 13565-905 São Carlos, SP, Brazil; ^4^Departamento de Fisiologia, Universidade Federal do Ceará (UFC), 60430-160 Fortaleza, CE, Brazil; ^5^Programa de Pós-graduação em Ciências Farmacêuticas, Faculdade de Farmácia, Universidade Federal do Pará (UFPA), Belém, PA, Brazil; ^6^Fundação Oswaldo Cruz (Fiocruz), 60180-900 Fortaleza, CE, Brazil; ^7^Unidad Académica Ciencias Químicas y la Salud, Universidad Técnica de Machala, 070151 Machala, Ecuador

## Abstract

The chemical composition and biological activity of a sample of yellow propolis from Mato Grosso do Sul, Brazil (EEP-Y MS), were investigated for the first time and compared with green, brown, and red types of Brazilian propolis and with a sample of yellow propolis from Cuba. Overall, EEP-Y MS had different qualitative chemical profiles, as well as different cytotoxic and antimicrobial activities when compared to the other types of propolis assessed in this study and it is a different chemotype of Brazilian propolis. Absence of phenolic compounds and the presence of mixtures of aliphatic compounds in yellow propolis were determined by analysing ^1^H-NMR spectra and fifteen terpenes were identified by GC-MS. EEP-Y MS showed cytotoxic activity against human tumour strain OVCAR-8 but was not active against Gram-negative or Gram-positive bacteria. Our results confirm the difficulty of establishing a uniform quality standard for propolis from diverse geographical origins. The most appropriate pharmacological applications of yellow types of propolis must be further investigated.

## 1. Introduction

Propolis is a beehive product popularly used to treat or prevent several disorders such as wound infections and respiratory conditions [1]. The regular intake of propolis has been indicated in traditional medicine as a way to promote health and enhance human resistance to infections or malignant affections with no unwanted side effects.

Brazilian propolis was previously classified by Park et al. [2] who described twelve distinct groups of Brazilian propolis. Green propolis (type 12) from south-eastern Brazil is currently the most exported Brazilian propolis. Green propolis is rich in artepillin C and other prenylated phenolic compounds with potent antitumour properties [3]. A brown type of propolis is found in the southern regions of Brazil (states of Paraná and Santa Catarina) [1, 4]. Yellow propolis samples from northeast and south Brazil were found by Park et al. [5] and classified in group 1 from southern region and groups 9 and 11 from northeast. In addition, yellow propolis from Cuba was described by Cuesta-Rubio et al. [6]. A 13th type of Brazilian propolis was later identified as the red propolis from northeastern Brazil [7]. Trusheva et al. [8] showed that this red propolis was rich in phenolics, triterpenoids, isoflavonoids, and prenylated benzophenones and a naphthoquinone epoxide was isolated for the first time from a natural source. Since then, red Brazilian propolis has been the target of further investigation by several research groups [6, 7, 9–13]. Sawaya et al. [4] have also carried out several studies seeking to classify the Brazilian propolis based on their ESI-MS fingerprints and chemometric multivariate analysis.

Despite the great number of studies about Brazilian propolis, the chemical composition of propolis from the central-western region of Brazil has been scarcely investigated [4, 19]. Neither the chemical composition nor the pharmacological properties of yellow Brazilian propolis have been described so far. In the current study, we investigated the chemical composition and cytotoxic and antimicrobial activities of a sample of yellow Brazilian propolis collected in the Pantanal ecosystem in Mato Grosso do Sul, Brazil. Moreover, four samples of typical Brazilian classes of propolis, green propolis from São Paulo (EEP-G SP), green propolis from Minas Gerais (EEP-G MG), red propolis from Bahia (EEP-R BA), brown propolis from Paraná (EEP-B PR), and a sample of yellow propolis from Cuba (EMP-Y Cuba), were included in the study, permitting the direct comparison with the chemical characteristics and pharmacological potency of yellow propolis.

## 2. Experimental

### 2.1. Propolis Samples and Extracts

Propolis was supplied by beekeepers from different states of Brazil: red propolis (Bahia), green propolis (São Paulo), yellow propolis (Mato Grosso do Sul), and brown propolis (Paraná). A sample of green propolis from the state of Minas Gerais was purchased in a local market. A sample of yellow propolis from Cuba was prepared as a methanol extract. All propolis samples were stored at −18°C until extraction. Ethanol extracts of propolis (EEP) were prepared using 10 g of each propolis samples mixed with 100 mL of absolute ethanol. The mixture was stirred for one day under controlled speed (160 rpm) at room temperature and then filtered. The filtrates were kept in freezer overnight (temperature of −18°C) and filtered again to remove waxes. Solvent from the extractive solutions was removed in a rotatory evaporator at 50°C to obtain the dry ethanol extracts of propolis (EEPs).

### 2.2. ^1^H-NMR Analysis

Samples of EEPs for ^1^H-NMR analysis were prepared as follow: a mixture of 100 *μ*L D_2_O (buffer phosphate pH 7.04) and 600 *μ*L CD_3_OD was added to 30 mg of propolis extracts and sonicated for 15 min. The mixture was centrifuged at 13000 rpm for 20 min at room temperature. The supernatant (600 *μ*L) was transferred into an NMR tube of 5 mm. Sodium-3-trimethylsilyl-2,2,3,3-d_4_ propionate (TMSP 0.324 mg/mL) was used as internal reference. All NMR experiments were recorded at 298 K in a Bruker UltraShielding*™* Plus 600 MHz spectrometer operating at 14.6 T, equipped with a TCI cryoprobe for H-C/N-D. ^1^H NMR spectra were acquired using a noesy pulse sequence for presaturation on water resonance and spoil gradient during mixing time (noesygppr1d, Bruker terminology). The parameters set in this sequence were 4.0 s for relaxation delay time, acquisition time of 3.99, data points of 140k, mixing time of 10 ms, and 128 scans with a spectral window of 30 ppm. Spectra were processed by applying an exponential line broadening LB of 0.3 and manually phased trough Topspin 3.0 (Bruker Biospin).

### 2.3. UPLC-ESI(−)-MS/MS

The EEPs were analysed on a UPLC Acquity Chromatographer (Waters, Milford, USA) coupled with a TQD Mass Spectrometer (Micromass-Waters Manchester, England), with an Electrospray source (ESI). Fingerprints were obtained by direct injection of 5 *μ*L of extracts by flow infusion into the ESI source. Chromatographic separation was carried out in a C_18_ BEH Waters Acquity analytical column (50 mm × 2.1 mm i.d., 1.7 *μ*m particle size) held at 30°C. A linear gradient elution was carried out with mili-Q purified water containing 0.1% formic acid (solvent A) and acetonitrile (solvent B) at a flow rate of 0.2 mL/min. Elution started with 95% of solvent A and 5% of solvent B. A linear gradient was performed until 100% of solvent B in 9 min and this condition was held for 1 min. Afterwards, the initial elution condition was reestablished in 2 min, totalizing 12 min for the chromatographic run. ESI(−)-MS and tandem ESI(−)-MS/MS were obtained under the following analytical conditions: capillary −3.5 kV, cone −30 V, source, and desolvation temperature were 150 and 350°C, respectively. For ESI(−)MS/MS, the energy for the collision induced dissociations (CID) was 25 V. Data were acquired in the* m/z* 100–700 range. Diagnostic ions in the different propolis samples were identified by the comparison of their ESI(−)MS/MS dissociation patterns with authentic analytical standards and/or by comparison with fragmentation pattern from the literature.

### 2.4. GC-EIMS

An aliquot of dry yellow propolis extracts was dissolved in ethyl acetate at a concentration of 500 *μ*g/mL. Volume samples of 1 *μ*L were injected in the splitless mode into an AGILENT gas chromatograph (model 7890A GC System), coupled with a mass spectrometer operating in EI mode at 70 eV. A 5% phenyl 95% dimethylpolysiloxane capillary column (30 m × 0.25 mm i.d., 0.25 *μ*m film thickness) was held at 250°C for 1 min and then heated to 300°C at the rate of 5°C/min. The final temperature was maintained for 10 min. Helium was used as the carrier gas. Injector and detector temperature was 230°C and 150°C, respectively. Compounds were identified by searching against a database of mass spectra (NIST 2011 Mass Spectral Library, Agilent Technologies).

### 2.5. Cytotoxicity Assays

The cytotoxicity of propolis extracts was evaluated against four human tumour cell lines: OVCAR-8 (ovary carcinoma), HCT-116 (colorectal carcinoma), SF-295 (human glioblastoma), and LH-60 (promyelocytic leukemia) obtained from the National Cancer Institute (Bethesda, MD, USA). The general viability of cultured cells was determined by the reduction of the yellow dye 3-(4,5-dimethyl-2-thiazolyl)-2,5-diphenyl-2H-tetrazolium bromide (MTT) to a blue formazan product, as previously described by Mosmann [20]. The tumour cells were maintained in RPMI 1640 medium, supplemented with 10% foetal bovine serum, 1% penicillin, and streptomycin at 37°C with 5% CO_2_. For all experiments, cells were seeded at 0.1 × 10^6^ cells/mL (LH-60, OVCAR-8, and SF-295) and 0.7 × 10^5^ cells/mL (HCT-116) and incubated during 72 h with propolis extracts at 37°C with 5% CO_2_. After centrifugation and solution removing, MTT solution was added and the plates were incubated and centrifuged and the solids dissolved in pure and sterile DMSO. The absorbance was measured in a plate spectrophotometer DTX-800 (Beckman Coulter) at 595 nm. Investigation of the survival viability for nontumour cell line L929 (mouse fibroblast) was also carried out. DMSO (solvent) and doxorubicin (reference standard drug) were used as negative and positive controls, respectively.

### 2.6. Antibacterial Activity

Antibacterial activity was evaluated against the following standard strains: (i) Gram-positive bacteria:* Staphylococcus aureus* (ATCC 6538) and* Enterococcus faecalis* (ATCC 29212) (ii) bacteria Gram-negative:* Pseudomonas aeruginosa* (ATCC 25853) and* Escherichia coli* (ATCC 8739). All samples were obtained from INCQS/FIOCRUZ (National Institute of Quality Control in Health, Brazil). Furthermore, one clinic isolate, methicillin-resistant* Staphylococcus aureus* (MRSA), was also applied as test organism and obtained from cultures of patient samples existing in the Public Hospital (Bacteriological Laboratory) of Belém city, Pará, Brazil.

All bacteria were previously seeded in Petri plates containing Mueller Hinton agar (Merck, Germany) and incubated at 35°C for 24 hours. For bacterial inoculum preparation, strains were grown to exponential phase in Mueller Hinton broth (Merck, Germany) at 37°C for 24 h and adjusted by diluting fresh cultures to turbidity equivalent to 0.5 McFarland scale (approximately 2 × 10^8^ CFU/mL) and then diluted until 1 × 10^3^ CFU/mL, as described by Clinical and Laboratory Standards Institute [21]. Minimum inhibitory concentration (MIC) and minimum bactericidal concentration (MBC) assays were performed using the broth microdilution method in MHB [21]. MIC is defined as the lowest concentration of extract with no visible growth of the microorganism in the resazurin colorimetric assay. To determine MIC, propolis extracts were dissolved in DMSO at the highest concentration (19000 *μ*g/mL) to be tested. A serial twofold dilution was made in a concentration range from 100 to 19000 *μ*g/mL in 1 mL sterile test tubes containing MHB. For the microdilution test, the inoculum (100 *μ*L) containing 5 × 10^3^ CFU/mL was added to each well and 100 *μ*L was transferred into consecutive wells. After 24 h of incubation, 15 *μ*L of resazurin (1 *μ*g/mL), which was metabolically reduced by active cells to a colour derivative, was added to the wells to allow visual identification of metabolic activity [22]. After incubation, the development of a purple-pink colour was considered as the indicative of bacterial growth. Therefore, MIC was read as the lowest concentration of the extract where the purple-pink colour was not observed. To determine MBC, 10 *μ*L of broth was taken from each well and incubated in Mueller Hinton agar at 37°C for 24 h and for each bacterium. The MBC was defined as the lowest extract concentration that resulted in a colony count lower than three colonies per mL (99.9% killing) or no bacterial growth, as described by de Quadros et al. [23]. Each test was performed in three replicates. Negative control consisted of 100 *μ*L of the bacterial inoculum and 100 *μ*L of DMSO. Chloramphenicol (50 *μ*g/mL) and gentamicin (10 *μ*g/mL) were used as positive controls for Gram-positive and Gram-negative bacteria, respectively.

### 2.7. Statistical Analysis

For chemometrics analysis of NMR a bucket table was created using AMIX Statistics software (version 3.9.7, Bruker Biospin). A bucket window of 0.04 ppm was choosen for spectral binning and residual water signal (4.90–5.00 ppm) and methanol (3.29–3.33 ppm) were excluded together with noise regions prior bucketing process. Principal component analysis (PCA) was used in order to compare the qualitative chemical composition of the diferent types of propolis extracts from several states of Brazil. Statistical differences between experimental types of propolis were verified by ANOVA followed by Fisher and Tukey test at 95% significance (*p* < 0.05).

## 3. Results and Discussion

### 3.1. Qualitative Comparison of the Chemical Composition of Propolis Extracts by ^1^H NMR, ESI(−)-MS, and LC-ESI(−)-MS/MS

**Figure 1:**
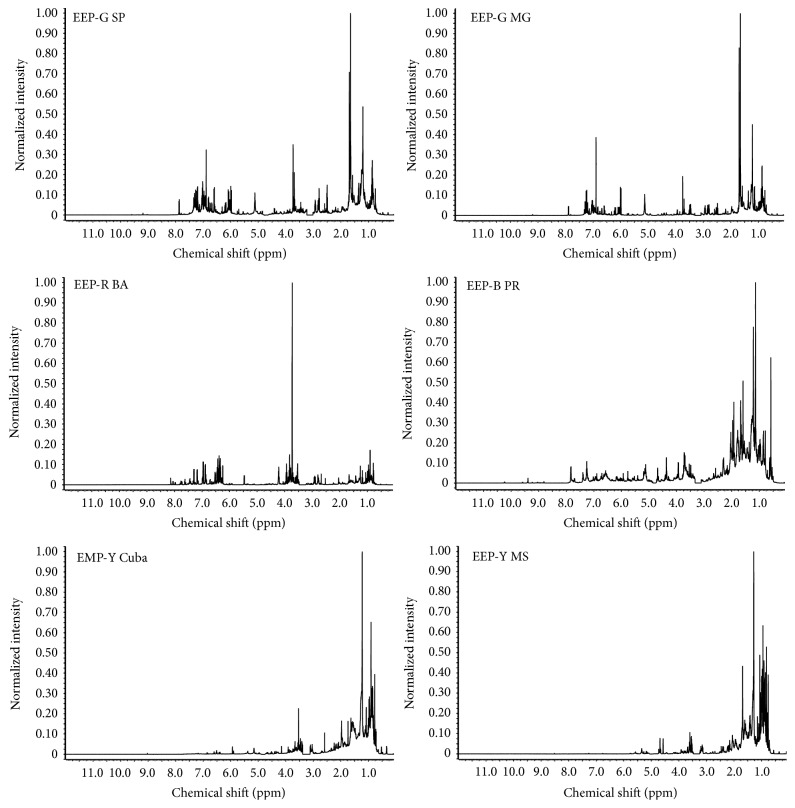
^1^H NMR (600 MHz, CD_3_OD/D_2_O) of propolis extracts (solvent signal at *δ* 3.36–3.27 and the signal for the internal reference TMSP were excluded from NMR spectra).

**Figure 2:**
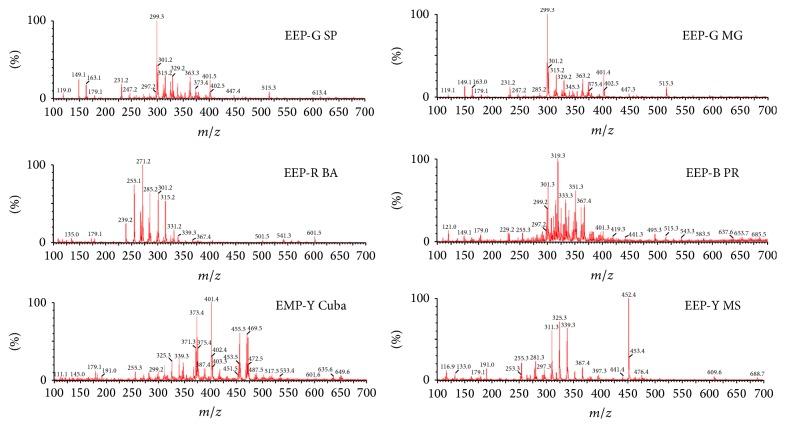
ESI(−)-MS fingerprints of propolis extracts.

The qualitative profile of green, red, brown, and yellow propolis from different regions of Brazil and one yellow propolis from Cuba was evaluated first by ^1^H NMR (Figure 1) and by ESI(−)-MS fingerprinting (Figure 2). The samples of Brazilian propolis showed clearly distinct ^1^H NMR (Figure 1) and ESI(−)-MS (Figure 2) patterns, indicating different chemical composition. The most striking feature in the ^1^H NMR spectrum of yellow propolis is the absence of signals from aromatic compounds and the most down field hydrogens are vinyl hydrogens at *δ* 5.0 to 6.0 ppm. This indicates the lack of abundance of phenolics compounds that are typically present in the green, brown, and red types of Brazilian propolis. In Figure 1, solvent signal (D_2_O/CD_3_OD) at 3.36–3.27 ppm and the signal for the internal reference TMSP were excluded from NMR spectra for better graphic quality.

**Figure 3:**
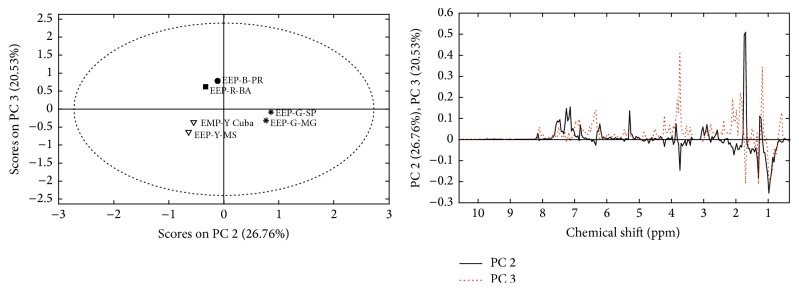
Principal component analysis of ^1^H-NMR data for extracts of propolis.

Through ^1^H-NMR technique, it is possible to verify the chemical class of only the most abundant compounds in the extracts, once NMR technique is quite susceptible to compound concentration. Chemometric analysis of ^1^H NMR data clustered propolis samples into three groups (Figure 3): a group comprising the green propolis; a second group for the brown and red propolis; and a third group for the yellow propolis. The Cuban and Brazilian yellow propolis were grouped due to the predominance of resonances at *δ* 0.7–1.1 ppm and *δ* 1.23–1.32 ppm characteristic of hydrogens bonded to* Csp* ^3^ in aliphatic compounds.

The six samples of propolis were also analysed by UPLC-ESI(−)-MS/MS to tentatively identify some of the ions observed by ESI(−)-MS fingerprinting (Figure 2). A total of twenty-nine known compounds were identified in green, red and brown Brazilian propolis and Cuban yellow propolis (Table 1). All these compounds have been previously identified in propolis and correspond to phenolic compounds, such as derivatives of benzoic and cinnamic acids, flavonoids and prenylated phenolic compounds, but labdanic terpenes were also found [3, 4].

### 3.2. Chemical Composition of Yellow Brazilian Propolis by GC-EIMS

**Figure 4:**
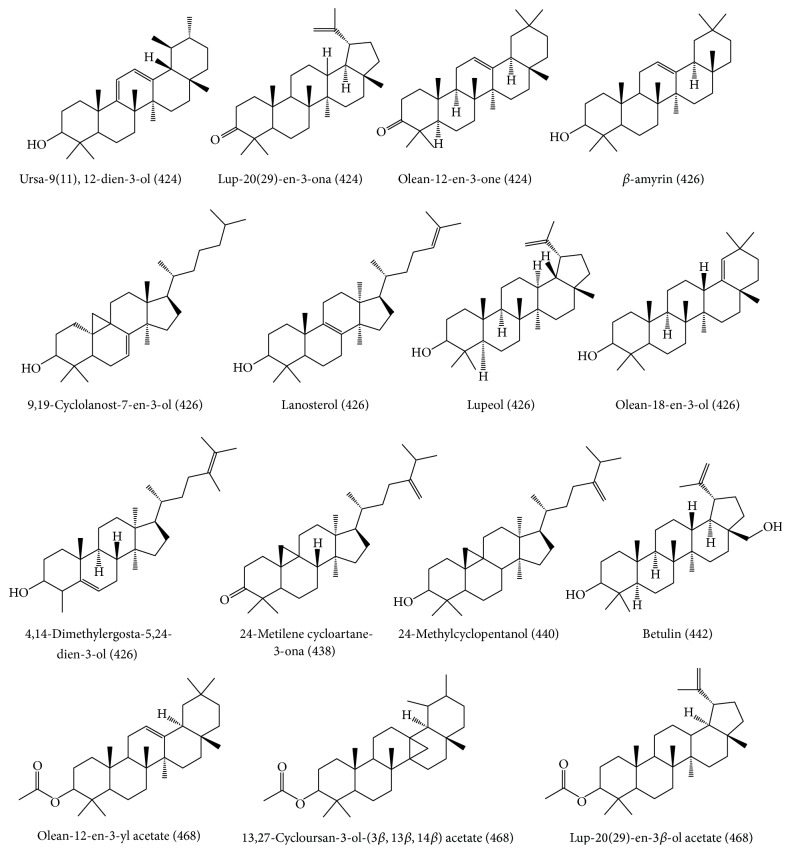
Compounds identified by GC-EIMS in the yellow propolis from Brazil.

The lipophilic nature of the constituents in yellow propolis made GC-MS the most suitable analytical technique to analyse the chemical composition of this type of propolis. A total of fifteen triterpenoids were tentatively identified in the extract of yellow propolis (Figure 4) through comparison of their fragmentation profile with data from the NIST library and the literature data. At least three triterpenoids were already reported for Brazilian type 6 propolis (red colour), such as *β*-amyrin, lupeol, and olean-12-en-3-one [8, 18, 24].

A study of the chemical composition of propolis produced in the state of Piauí (Brazil) resulted in the identification of six triterpenoids derived from cycloartane: isomangiferolic acid, mangiferolic acid, mangiferonic acid, ambonic acid, ambolic acid, and 24-methylene cycloartane-3*β*,26-diol. The authors mentioned that these compounds were previously isolated from the stem bark of species of* Mangifera indica* (Anacardiaceae) and suggested that this species could be the source of propolis from Piauí. Pentacyclic triterpenoids, such as lupeol, *α*-amyrin and *β*-amyrin, and tetracyclic cycloartenol type, have shown significant anti-inflammatory activity [25].

Hernández et al. [26] reported a quali-quantitative GC-MS study of 19 samples of yellow Cuban propolis. Yellow Cuban propolis comprised two major groups: type A, rich in triterpenic alcohols and with the presence of polymethoxylated flavonoids as minor constituents, and type B, containing acetyl triterpenes as the main constituents [6]. Through GC-MS compounds of low polarity were identified such as lanosterol, *β*-amyrone, *β*-amyrin, germanicol, lupeol, cycloartenol, *β*-amyrin acetate, 24-methylene-9,19-ciclolanostan-3*β*-ol, *α*-amyrin acetate, and lupeol acetate [26]. In our current report, all these compounds were found in the sample of yellow propolis from Pantanal (Mato Grosso do Sul, Brazil) demonstrating a similar chemical profile for the Cuban and Brazilian yellow propolis samples, which consequently formed a group in PCA (Figure 3).

### 3.3. *In Vitro* Cytotoxicity Assays

In preliminary screening of cytotoxic activity, the percentage of inhibition on three tumour cell lines was measured for each type of propolis (data not shown). Green samples (EEP-G SP and EEP-G MG) had low inhibition of all studied tumour cell lines, presenting low cytotoxic potential. Brown (EEP-B PR) and red (EEP-R BA) propolis had the highest cytotoxic potential with inhibition percentages greater than 75% in at least two tumour cell lines. Yellow propolis from Brazil (EEP-Y MS) showed high cytotoxic activity only against OVCAR-8 tumour cell, thus showing greater specificity against ovarian carcinoma.

Only extracts of red, brown, and yellow propolis, which showed inhibition percentages above 75% in at least one tumour cell, were considered for further experiments. An analytical curve obtained by linear regression, varying the concentration of extract of propolis and measuring inhibition of cell proliferation, enabled the calculation of the concentration required for each extract to inhibit 50% (IC_50_) of tumour cells OVCAR-8 (ovary carcinoma), HCT-116 (colorectal carcinoma), SF-295 (human glioblastoma), and LH-60 (promyelocytic leukemia) (Table 2). The highest concentration tested for each extract was 100 *μ*g/mL.

According to criteria established by the National Cancer Institute (NCI, USA), the IC_50_ threshold for extracts with promising cytotoxic activity value is 30 *μ*g/mL. The results showed that yellow propolis contains substances with cytotoxic effects. Brown propolis was effective against the four cell lines studied. To evaluate the degree of selectivity against tumour cell lines, IC_50_ was also investigated against the nontumour cell line L929 (mouse fibroblast) and a selective index (SI) was calculated as IC_50_ (nontumour cell)/IC_50_ (tumour cell). The highest selectivity against all tumour cells was shown by red propolis especially against LH-60 (leukemia promyelocytic). da Silva Frozza et al. [17] also found that red propolis had selective cytotoxic activity for tumour cell lines. Yellow propolis had selective indexes between 0.91 and 1.84; however, only values of SI ≥ 2 are considered significant [27]. Therefore, our results suggest that brown and red propolis and, to a lesser extent, yellow propolis may act in a selective way against tumour cells and show potential antitumour activity. We previously demonstrated that propolis extracts showed an* in vivo* antitumour activity in the experimental model Sarcoma 180 tumour cells with moderate toxicity effect at experimental exposure levels when compared to 5-FU [16]. Propolis has been a subject of intensive research, especially in the area of cancer, and its selectivity* in vivo* against tumours must be further investigated.

### 3.4. Antibacterial Activity

Samples of green (EEP-G MG and EEP-G SP) and red propolis (EEP-R BA) had high antibacterial activity against Gram-positive bacteria, mainly* S. aureus* (Table 3). Particularly, EEP-G SP showed excellent activity against* S. aureus* (MIC = 159 *μ*g/mL),* E. faecalis* (MIC = 310 *μ*g/mL), and MRSA isolate (MIC = 630 *μ*g/mL).

As previously reported, the antimicrobial activity and chemical composition of propolis are directly associated with geographical location, biodiversity, bee species, and method and time of harvest [28, 29]. The high antibacterial activity of propolis against Gram-positive bacteria, mainly strains of* S. aureus* and* Enterococcus* sp., has been widely described [29–31]. However, previous studies have shown a wide variation in MIC and MBC values [29, 31]. Up to now, there are few studies on the antimicrobial activity of green and red propolis. A study of Brazilian red propolis from state of Alagoas reported that red propolis inhibited* Streptococcus pyogenes* and various Gram-negative bacteria, such as* P. aeruginosa* and* E. coli* with MIC values of 256 to 512 *μ*g/mL [18]. Another study about Brazilian red propolis from the state of Alagoas reported MICs values of 50 to 100 *μ*g/mL against* S. aureus* ATCC 25923 and* Streptococcus mutans *UA159 [10]. Regarding green propolis types, a propolis sample collected in São Paulo state inhibited* S. mutans* at concentrations of 400 *μ*g/mL [32]. If compared with previous studies, our results showed that green propolis from São Paulo and Minas Gerais states was more effective at inhibiting Gram-positive bacteria, with MIC values of 159 to 310 *μ*g/mL, whereas Brazilian red propolis from Bahia state showed antibacterial action similar to other studies published. The higher antibacterial activity expressed by Brazilian green propolis may be attributed to its different chemical composition and its high concentration in flavonoids and aromatic acids such as galangin, kaempferol, pinostrobin, and pinocembrin which have shown high antibacterial effect, as reported by Grenho et al. [33]. In addition, Scazzocchio et al. also reported that some constituents such as flavonoids (kaempferide, quercetin, galangin, and pinocembrin), caffeic, benzoic, and 4-hydroxy-3,5-diprenylcinnamic acids may probably act by the inhibition of bacterial RNA polymerase but may also act on the local microbial membrane or cell wall, causing structural and functional damage, resulting in antimicrobial action [30].

On the other hand, our data showed that the two yellow propolis samples (EEP-Y MS and EMP-Y Cuba) were not active against Gram-negative bacteria or Gram-positive bacteria, with MIC values above 5020 *μ*g/mL. So far, there are no previous reports on the chemical composition of yellow propolis from Brazil and reports about their biological activities are also scarce. Park et al. [5] reported that samples of yellow propolis from Brazil were not active (or weakly active) against* S. aureus*. Additionally, yellow propolis samples had low antioxidant and anti-inflammatory activities. On the other hand, a previous report about yellow propolis from Cuba showed that extracts were able to inhibit 50% of* S. aureus* growth at low concentration, but no activity was observed against* E. coli* [34]. These yellow propolis samples from Cuba were abundant in triterpenoids and had a small proportion of phenolics and flavonoids if compared with green and red propolis.

## 4. Conclusions

The chemical composition of a yellow propolis from Mato Grosso do Sul/Brazil was analysed and its* in vitro* biological activity was assessed for the first time. This sample is rich in triterpenes and presents a different qualitative profile from other well-known types of Brazilian propolis. Yellow propolis showed cytotoxic activity against human ovarian carcinoma but was not active against Gram-negative or Gram-positive bacteria. Our results indicate that the Brazilian brown, red, and, to a lesser extent, yellow propolis inhibited, in a selective way, the growth of tumour cells and therefore show potential for anticancer therapy. Brazilian green propolis showed better antibacterial action, mainly against* S. aureus* and one multidrug-resistant clinical isolate (MRSA).

The results of the present study expand the knowledge about the chemical composition and biological activities of different chemotypes of propolis from Brazil, showing its variability and difficulty of standardization. Additionally, there is a need to investigate the most appropriate pharmacological applications for the yellow type of propolis due to its unique composition when compared to other types of Brazilian propolis.

## Figures and Tables

**Table 1:** Propolis constituents identified by UPLC-ESI(−)-MS/MS.

*m*/*z* [M-H]^−^	Compound	Propolis origin	ESI(−)-MS/MS *m*/*z* (relative intensity%)	Reference
**151**		SP	150 (100); 147 (65); 127 (10); 121 (15)	
**163**	*p*-Coumaric acid	SP, Cuba, MG, PR	163 (20); 119 (100)	a, [4, 14, 15]
**179**	Caffeic acid	SP, MG, PR	179 (40); 135 (100); 116 (50)	[14]
**229**	2,2-Dimethyl-6-carboxyethenyl-2H-1-benzopyrane	SP, MG	185 (100); 168.9 (50); 146 (20)	a, [4]
**231**	4-Hydroxy-3-prenylcinnamic acid	SP, MG, PR	231 (15); 187 (30); 132 (100)	a, [4, 15, 16]
**233**	Viscidone	SP, MG, PR	233 (25); 188 (100); 133 (50); 132 (85)	a
**247**	3,4-Dihydroxy-5-prenylcinnamic acid	SP, MG, PR	247 (40); 203 (60); 148 (100)	a, [16]
**253**	2-Hydroxy-4- methoxychalcone	BA	253 (80); 237 (95); 209 (100); 161 (70); 136 (92)	[17]
**253**		SP, MG	220 (5); 162 (20), 145 (30); 118 (100)	
**255**	Liquiritigenin	BA	255 (35); 135 (40); 119 (100)	[7, 9, 17, 18]
**255**		BA, Cuba	255 (45); 135 (35); 119 (100)	
**255**		MS	254 (52); 209 (100); 191 (50); 153 (62); 123 (62); 109 (61)	
**285**	(*3S*)-Vestitone	BA, MG	285 (25); 269 (33); 147 (18); 109 (100)	[18]
**285**	Pinobanksin-5-methyl-ether	SP, MG	285 (100); 252 (30); 163 (45); 151 (35); 136 (50)	[14]
**285**	Kaempferol	Cuba, BA	285 (83); 149 (100); 122 (85)	[14]
**297**		BA	267 (85); 205 (100)	
**299**		BA	299 (40); 284 (100)	
**299**	Luteolin-methyl-ether	BA	299 (40); 284 (100); 255 (25); 227 (30)	[14, 15]
**299**	Kaempferide	SP, Cuba, PR	299 (100); 284 (90); 200 (30); 151 (23)	[15, 16]
**299**	Artepillin C	SP, MG, PR	299 (90); 255 (100); 200 (97)	a, [4, 16]
**301**	Dihydrokaempferide	BA, SP, Cuba, MG, PR	301 (100); 283 (5); 152 (30)	a, [16]
**315**	(3*S*)-Violanone	BA	315 (20); 108 (100)	[18]
**315**	Quercetin-3-methyl-ether	BA	315 (80); 300 (55); 271 (50); 243 (100); 165 (48)	a, [14]
**315**	Isorhamnetin	SP, MG, PR	315 (100); 284 (20); 252 (30)	a, [15]
**315**	(3-4-Hydroxy-3-methyl-2-butenyl-4-hydroxy-5-prenyl) cinnamic acid	SP, MG	315 (100); 253 (90); 241 (88); 198 (98)	a
**315**	3-Hydroxy-2,2-dimethyl-8-prenylchromane-6-propenoic acid	SP, MG	315 (75); 271 (25); 253 (20); 244 (85); 198 (100); 146 (45)	[15]
**317**		BA	317 (20); 270 (23); 166 (48); 109 (100)	
**317**		SP, MG	317 (25); 314 (50); 241 (100); 124 (73)	
**317**		SP, MG	317 (10); 242 (40); 200 (100)	
**317**		SP	317 (45); 273 (55); 160 (100)	
**317**		MG	317 (40); 312 (100); 253 (50); 147 (80)	
**319**		BA, PR	301 (100); 193 (25)	
**319**		MS	319 (20); 223 (100);	
**329**		BA	329 (30); 314 (55); 299 (100); 285 (35); 271 (100); 182 (60)	
**329**	Quercetin-dimethyl-ether	SP, Cuba, MG, PR	229 (100); 314 (70); 299 (42); 270 (80); 227 (25)	[14]
**329**	Betuletol	SP, MG	329 (85); 314 (45); 299 (100); 270 (85); 257 (90) 198 (50); 160 (35)	a
**333**	Agathic acid	SP, MG, PR	333 (45); 314 (40); 257 (30); 245 (35)	a
**347**	Agathic acid 15-methyl ester	SP	247 (70); 259 (20); 187 (100); 163 (60); 146 (80)	a
**353**		Cuba, MS	353 (40); 335 (45); 151 (40); 112 (50)	
**361**	15-Acetoxy cupressic acid	MG, SP	361 (100); 317 (70); 242 (75); 159 (45); 126 (72)	a
**363**	3-Prenyl-4-dihydrocinnamoyloxy cinnamic acid	SP, MG	363 (20); 187 (80); 149 (100)	a, [15]
**447**	(*E*)-3-{-4-hydroxy-3-[(*E*)-4-(2,3-dihydrocinnamoyl oxy)-3-methyl-2-butenyl]-5-prenylphenyl}-2-propenoic acid	SP, MG, PR	447 (10); 297 (50); 197 (15); 149 (100)	a, [16]
**515**	3,5-DI-*O*-caffeoylquinic acid	SP, MG, PR	515 (25); 353 (100); 173 (30)	a, [4, 15]

^a^Identification based on comparison with authentic analytical standards.

**Table 2:** IC_50_ (*µ*g/mL) and selectivity index (SI) for brown, red, and yellow propolis extracts against a panel of tumour cells and against a normal cell L929.

Tumour cells									Nontumour cells
	SF-295		HCT-116		OVCAR-8		HL-60		L929
	IC_50_	SI	IC_50_	SI	IC_50_	SI	IC_50_	SI	
EEP-BPR	27.91 (26.88–28.98)	7.77	19.43 (16.52–22.84)	11.17	26.97 (25.81–28.19)	8.05	9.44 (8.67–10.28)	22.99	217 (101.9–461.8)
EEP-RBA	70.66 (9.06–551.0)	20.20	42.53 (14.93–121.2)	20.20	53.46 (44.30–64.52)	16.07	17.48 (14.66–20.85)	49.15	859.1 (137.5–5368)
EM-YMS^*∗*^	16.4 (11.6–23.1)	1.84	43.73 (32–59)	0.69	20.67 (18.7–38)	1.46	31.56 (22.4–44.4)	0.95	30.11 (23.45–38.66)
EEP-YMS	34.77 (25–48.3)	0.91	>50	—	17.9 (17.63–52.65)	1.70	>50	—	31.58 (21.29–46.87)
Dox	0.145 (0.127–0.162)	2.71	0.063 (0.046–0.081)	6.03	0.26 (0.16–0.30)	1.46	0.011 (0)	34.54	0.38 (0.28–0.48)

^*∗*^Only for yellow propolis an extract was also obtained with methanol as extractive solvent.

**Table 3:** Minimum inhibitory concentration (MIC) and minimum bactericidal concentration (MBC) for different extracts of propolis.

Propolis	Bacteria, MIC and MBC/(*µ*g/mL)									
	*Staphylococcus aureus*		Methicillin-resistant *S. aureus (MRSA)*		*Enterococcus faecalis*		*Pseudomonas aeruginosa*		*Escherichia coli*	
	MIC	MBC	MIC	MBC	MIC	MBC	MIC	MBC	MIC	MBC
EEP-Y MS	>12,800	>12,800	>12,800	>12,800	>12,800	>12,800	6,400	>12,800	>12,800	>12,800
EMP-Y Cuba	>10,500	>10,500	>10,500	>10,500	5,020	10,500	5,020	6,400	>10,500	>10,500
EEP-B PR	>19,900	>19,900	>19,900	>19,900	860	>1,730	>13,900	>13,900	>13,900	>13,900
EEP-G MG	200	400	400	>400	400	800	6,450	12,900	6,450	12,900
EEP-G SP	159	315	630	>630	310	>630	10,110	20,220	10,110	20,220
EEP-R BA	390	780	780	>780	780	1,570	6,300	12,600	6,300	12,600
Chloramphenicol	50	>50	>50	>50	50	>50	—	—	—	—
Gentamicin	—	—	—	—	—	—	4	10	10	10
DMSO	>19,900	>19,900	>19,900	>19,900	>19,900	>19,900	>19,900	>19,900	>19,900	>19,900
